# Mr. Hill, on Angina Pectoris

**Published:** 1800-07

**Authors:** G. N. Hill

**Affiliations:** Chester


### *To the Editors of the Medical and Phyfical Journal.*

Gentlemen,

Dr. Black, in his inftructive paper on the Angina Pectoris, Art. XX. Vol. IV. of the Memoirs of the Medical Society of London, obferves, "Diffections have fhewn this difeafe to be connected, either with fome organic degeneracy of the heart or great veffels, or with fome mechanic preffure upon them, arifing either from an affufion of fluids, or an accumulation of fat;"

fat;" and concludes with the following query, "Are women exempted from this diseafe? I do not recollect any decided inftance on record of its occurring in the female fex; and fome of the oldeft and moft experienced phyficians in this part of the kingdom have made the fame remark."

The following cafe, recently under the care of Dr. Arden, of this city, and myfelf, will, I hope, be acceptable to the readers of your ufeful Journal.

Your obedient fervant,

*Chefter, May* 12, 1800. G. N. HILL.

---

Mrs. E. B. from infancy up to the age of twenty-two, of a fpare habit of body, fubject to few complaints, excepting a periodical hemicrania of the left fide, accompanied with copious bilious vomitings; from a change of her fituation in life, which had hitherto been very abftemious, to full living on animal food, and the liberal ufe of malt liquors, became fuddenly very fat; to obviate which, recourfe was had to fevere labour, acids, and ftarving, with a total abftinence from all drinks heretofore ufed. An increafe of the hemicrania, violent dyfpeptic fymptoms, irregularity of the menfes, and confiderable emaciation, were the confequences: Removal to a more healthy air, change of diet, and moderate labour, removed thefe affections; and, excepting occafional recurrence of the affection of the face, Mrs. B. enjoyed tolerable health, until the age of thirty-three; at this period, from the concurrent caufes of eafe, indolence, full meals of animal food, particularly late fuppers, the ufe of malt liquors, indulgence in fleep after dinner, and almoft total neglect of exercife, obefity returned with quick fteps, and produced an alarming change. One day, after eating a hearty dinner very eagerly, whilft attempting to walk to a neighbouring village, fhe was in a moment feized with a violent pain under the left breaft, extending acrofs the fternum, towards, but not quite reaching, the right fide; it compelled her to ftop inftantly, when refting on a ftone, it went off in a few minutes. Though very much frightened, yet, being a woman of great refolution, fhe perfifted in compleating her walk, notwithftanding three or four fimilar attacks before reaching home; but, owing to the precaution of walking very deliberately, and refting often, none of thefe equalled the firft in violence or duration. She defcribed the pain as attended with great and inconceivable diftrefs at her breaft, as though her heart gave a violent ftroke upward towards her throat, and then fell down again, at which inftant the anxiety was indefcribable, but commonly relieved for a moment ment by expulſion of wind from the ſtomach, and a deep lengthened ſigh. The pain for ſome months, or near two years, never recurred but upon uſing ſudden or violent exerciſe; and then, if any friend urged her to come on, and ſtrive againſt it, her apprehenſions were, that death muſt be the conſequence of the attempt. The obeſity continued increaſing, with the addition of a troubleſome incubus; at length, the pain occurred after eating heartily a favourite dinner and lying down on the bed, and often about midnight; hence, it became neceſſary a friend ſhould watch the approach of both theſe unhappy affections, and awake her; when ſhe would inſtantly get up, labouring under the apprehenſion of immediate diſſolution. The rather ſudden death of her huſband one morning very early, on receiving the intelligence, was ſucceeded by the pain, like a ſudden ſtroke or blow received on the left breaſt, and the firſt fainting fit; on recovering from which, ſhe complained of pain down her arm, and numbneſs in the hand, particularly the thumb, the whole limb being cold and covered with a clammy moiſture, as, indeed, was the whole body. I was preſent when this happened; on examining the pulſe, I found it very feeble and irregular, giving one weak ſtroke, and then two ſcarce perceptible; it was ſome time before it became regular, and remained feeble and fluttering all day. I preſcribed the camphor julap, made with peppermint water and a portion of æther, which ſtaid on her ſtomach, notwithſtanding a deadly ſickneſs, though not amounting to vomiting, being preſent. The medicine occaſioned the eſcape of much wind, and thereby afforded relief; a large bliſter to the breaſt, ſaline aperient medicines, thin weak liquids for drink, and an abſtemious diet for a few weeks, rendered her free from the faintings, and perceptibly reduced the corpulency. Neglect of this ſalutary plan, towards the latter end of laſt year, was followed by a ſevere attack in the night, ſoon after eating a hearty improper ſupper near twelve o'clock, after having juſt dropped aſleep; about two o'clock, a ſecond; and during my attendance with Dr. Arden, (now called in) a ſucceſſion of paroxyſms at irregular periods for ſeveral weeks: ſome of theſe were ſlight compared to others. Univerſal coldneſs, clammy ſweats, loſs of all ſenſe, and the power of voluntary motion, accompanied the more ſevere fits; and I often obſerved, that immediately on recovery from one, our patient would ſtretch herſelf upwards, ſtrive to yawn, throw her head back, and ſay, ſhe wiſhed ſhe had a rope fixed to a beam to pull at, believing it would expand her cheſt, and give her heart more room to beat. Saline, ſudorific, and gently aperient medicines were preſcribed, with low diet, though indeed, little now ſufficed, the conſtant constant distress of mind, labouring under continual apprehensions of death, seemed to suspend appetite: the tendency to become thinner soon evinced itself. Two large issues were now cut, one below each knee, the discharge from which being copious, afforded considerable relief; occasional moderate evacuations, with this constant drain, have reduced Mrs. B's size very considerably; her health has proportionally amended, but she is still subject to returns of the pain upon any sudden exertion of body, or particular emotion of mind, although it is now but rarely accompanied by fainting.

Dr. Parry, in his recent treatise on this formidable disease, when enumerating its symptoms, says, "And is altogether so free and distinct from any difficulty of breathing." Now, in the case I have recited, though not before mentioned, this symptom was most urgent, but only prevailed when Mrs. B. was using exercise: in those attacks of the disease which occurred when at rest, this symptom never appeared; but the deep sighing peculiar to such patients was now most conspicuous, accompanied with a lifting up of the arms, and an intolerable desire to expand the breast. From all which has now been said, permit me to add, I think there can be no doubt, but sudden increase of fatness is capable of proving a very alarming cause of angina pectoris; it is probable, that the four cases mentioned by Dr. Darwin in his Zoonomia, as cured by issues, abstemiousness, and antimonials, were of this description; an encouraging circumstance to those who shall be so unfortunate as to be afflicted with this disease in early life; I argue thus, because the complaint, when occurring in persons at a more advanced period, seems, from the clear deductions of Dr. P. to arise from ossification of the coronary arteries; and, unhappily, these curative means cannot merely be but little relied on, but, perhaps, would have a tendency rather to aggravate than relieve, inasmuch, as from our knowledge of the functions of the absorbent system, evacuating remedies tend powerfully to promote absorption; hence, may quicken, rather than retard the progress towards ossification. Dissections seem to have proved, that persons liable, or who have fallen victims to angina pectoris, are peculiarly inclined to fatness of the heart; in those, therefore, who acquire sudden, enormous, and distressing obesity, can we wonder the disease should occur so formidably? I have never met with a case of angina in advanced life, as described by authors on the subject; but I have some reasons to believe, it has occurred in very corpulent subjects, and been over-looked; one person in particular, a woman, I remember to have been so similarly affected to Mrs. B.' as, that the description of the one case might serve for that of the other, with this difference only, as to the event, that this patient perfifted in rejecting the ufe of all means prefcribed for her relief, and continuing to grow fatter daily, was one morning found dead in her bed, after having eaten a hearty fupper of animal food.

